# Bio-Based, Degradable, and Tunable Epoxy Thermosets from Homogenized Acids and Epoxidized Linseed Oil or Glycerol Triglycidyl Ether

**DOI:** 10.1021/acssuschemeng.5c10683

**Published:** 2025-12-17

**Authors:** Benjamin J. Groombridge, Gavin R. Irvine, Vlad Jarkov, Jonathan T. Husband, Strachan N. McCormick, Matthew G. Davidson

**Affiliations:** Institute of Sustainability and Climate Change and Department of Chemistry, 1555 University of Bath, Bath BA2 7AY, United Kingdom

**Keywords:** sustainable, bioderived, catalyst-free, epoxy materials, citric acid, green materials, polymers

## Abstract

Epoxy thermosets continue to be seen as desirable materials for high-performance applications, given their excellent thermomechanical properties. However, commercial epoxides are generally derived from non-renewable feedstocks and are highly resistant to chemical decomposition. Accordingly, the provision of readily degradable, bioderived alternatives offering both high performance and tunable thermomechanical properties is urgently necessary for the development of sustainable composite materials. In this work, such materials are prepared by the reaction of epoxidized linseed oil (ELO) with different quantities of organic acid hardeners, comprising eutectic mixtures of (trifunctional) citric acid (CA) and various concentrations of two different linear aliphatic diacids, pimelic acid (PA), and glutaric acid (GA). Variation of both acid chain length and diacid:triacid ratio permits controlled manipulation of the resulting materials’ thermomechanical properties, with extensive cross-linking in high-triacid systems yielding increases in both glass transition temperature and mechanical strength. The presence of diacid species ensures homogeneity of the reaction mixture during resin curing, without requiring exogenous solvents or other diluents to solubilize the CA, and all materials are shown to be readily degradable under aqueous basic conditions. Finally, preliminary studies indicate that replacement of ELO with glycerol triglycidyl ether (GTE) yields materials with further enhanced thermomechanical properties comparable to *Bisphenol* A diglycidyl ether-derived materials.

## Graphical abstract

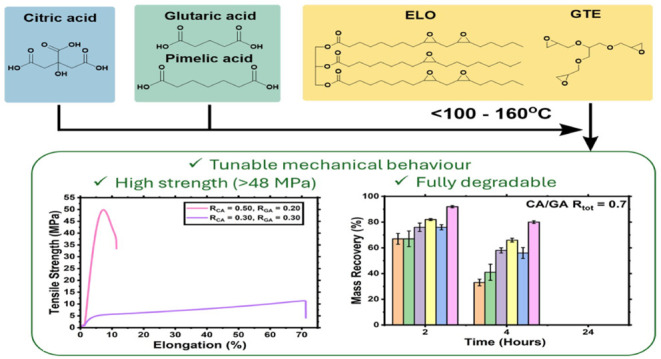

## Introduction

Epoxy thermoset materials have diverse applications at a range of scales, including in composite production, construction (e.g., flooring), coatings, and adhesives. These applications are enabled by their tunable, and often remarkable, thermal and mechanical properties, chemical inertness, and capacity for facile curing.^1–4^ However, the production of epoxy thermosets typically involves the use of hazardous monomers or monomer precursors derived from fossil resources.^5^ The extreme chemical resistance of these materials also presents challenges for end-of-life management,^6,7^ this being particularly pressing with the widespread and increasing use of carbon fiber-reinforced composite (CFRC) materials across sectors such as renewable energy and aerospace. The current state of the art for valorization of CFRC wastes involves pyrolytic or mechanical destruction of the epoxy matrix, or aggressive chemical treatments,^8,9^ to facilitate recovery of the fibers.^2,8^ Realizing circularity and sustainability in CFRC and other epoxy resin-based materials is reliant upon provision of bio-based and readily degradable alternatives with otherwise comparable, and similarly versatile, properties.

The preparation of epoxy thermosets requires a monomer containing several epoxide groups capable of undergoing ring-opening.^10^ In commercial epoxy thermosets, this is frequently a fossil-derived species such as bisphenol A diglycidyl ether (BADGE).^1,10,11^ An attractive source of alternative, bio-based monomers is the epoxidation of unsaturated vegetable oils *via* environmentally benign chemical treatments,^4^ with epoxidized soybean and linseed oils (ESO and ELO, respectively) being already commercially available. Another monomer of interest, which may be partially or wholly bioderived, is glycerol triglycidyl ether (GTE).^12^ Polymerization to afford an epoxy thermoset is readily effected by combining the epoxide-containing monomer with a hardener (comonomer) containing, for example, several amine or anhydride moieties,^13–19^ frequently in the presence of a suitable catalyst. However, a wide range of functional groups will readily react with epoxides, providing more sustainable alternatives to such established approaches.^20^ These include alcohols, carboxylic acids, and thiols,^20–30^ in addition to (catalytic) epoxide homopolymerization.^31–33^ Accordingly, bio-based species containing multiple carboxylic acid moieties provide a rich source of prospective comonomers for sustainable epoxy thermoset synthesis.

The thermal, mechanical, and, in the context of composites, interfacial properties of epoxy thermosets are significantly influenced by their cross-link density. Increased cross-linking generally yields harder, stiffer, but more brittle materials, with correspondingly higher glass transition temperatures (*T*_g_).^34–37^ The potential cross-link density is determined by the number of reactive moieties present per molecule of each monomer, with the use of exclusively bifunctional monomers affording only linear polymer chains. Accordingly, the properties of epoxy thermosets based on epoxidized vegetable oils (EVOs) can be manipulated by selection of both EVOs and acid-based hardeners with varying degrees of functionality.^36,38–40^ Specifically, the use of diacid and triacid comonomers, including their combination in various ratios, may permit the delivery of materials with diverse, finely controllable properties.

Citric acid (CA) represents a convenient bioderived triacid, commercially available at large scale, and presenting acceptably low ecological and toxicological hazards.^41^ However, its polar and highly crystalline nature precludes homogenization when combined with nonpolar EVOs.^42–44^ Nonetheless, a range of approaches has been reported to overcome this limitation, including the addition of water^42,45^ or other solvents,^43,46,47^ although these must then be removed from the curing thermoset, presenting process design challenges and potentially leading to changes in material volume during curing (void formation). Alternatively, nonvolatile, nonreactive eutectic components can be incorporated,^12,13,44,46,48^ or techniques such as ultrasonication^9^ or ball milling^14^ employed to form a homogeneous preresin. Various bioderived, linear aliphatic diacids are, likewise, commercially available, with glutaric (GA) and pimelic acids (PA), C5 and C7 species, respectively, being studied in this work. These acids were chosen as they can homogenize well with CA at moderate temperatures (forming a eutetic mixture), and they were expected to introduce some flexibility into the final thermoset. Collectively, these methods have been reported to afford CA and EVO-based epoxy thermosets that exhibit a wide variation in thermomechanical properties, described by their *T*_g_ and Young’s moduli values (*E*, Table 1).^49^

**Table 1:** Preresin and Curing Conditions for the Synthesis of EVO-Citric Acid Thermosets alongside Their Resulting *T*_g_ and Young’s Modulus (*E*) Values as Reported by Various Research Articles

EVO^a^	COOH/epoxy	Preresin conditions	Curing conditions	*T*_g_ (°C)	*E* (MPa)	Reference
ECO	1	THF	1. 90 °C, 6h 2. 120 °C, 2h	38	0.26	Sahoo 2018 ^50^
ESO	1	Water	95 °C, 24h	–2	1.43	Hood 2022 ^51^
ESO	1.1	Ultrasonication	120 °C, 5h	26	2.10	Moser 2024 ^52^
EPO	1.1	Ultrasonication	120 °C, 5h	22	1.80	Moser 2024 ^52^
ELO	1	Water	1. 90 °C, 3h 2. 120 °C, 1h 3. 150 °C, 1h	39	37	Necolau 2022 ^47^
ELO	1	THF	1. 90 °C, 3h 2. 120 °C, 1h 3. 150 °C, 1h	41	120	Necolau 2022 ^47^
ELO	1	THF	1. 90 °C, 6h 2. 120 °C, 2h	43	54	Sahoo 2018 ^50^
ELO	0.8	Ethyl lactate (1)	1. 60 °C 1h, 2. 160 °C, 2h	35	33	Tellers 2020 ^46^
ELO	0.8	Ethyl lactate (0.5)	1. 60 °C 1h, 2. 160 °C, 2h	40	630	Tellers 2020 ^46^
ELO	0.8	Diethyl malonate (1)	1. 60 °C 1h, 2. 160 °C, 2h	40	101	Jamali-Moghadam-Siahkali 2024 ^53^
ELO	0.76	Ball mill, anti bubbling agent	1. 80 °C, 24h 2. 120 °C, 24h	82	1100	Anusic 2020 ^54^

a ECO = Epoxidized Castor Oil, EPO = Epoxidized Pennycress Oil.

Herein, we report the facile use of eutectic mixtures comprising CA and, variously, GA and PA, as bio-based hardeners for ELO, affording a family of fully bio-based thermosets. Crucially, this has been achieved in the absence of any unreactive components such as solvents, catalysts, or other additives, as well as tricky to scale techniques such as ball milling.

## Experimental Section

### Synthesis of Epoxy Thermosets

For all epoxy thermosets, the ratio of carboxylic acid to epoxide groups was expressed as *R*_tot_ where *R*_tot_ = [COOH]/[epoxide] (see eq 1). For calculation of this value, the numbers of carboxylic groups from each hardener, relative to the number of epoxide groups present, are considered separately with the total number of carboxylic groups being equal to their sum. The values associated with each constituent hardener appear as *R*_CA_ for citric acid content, *R*_GA_ for glutaric acid content, and *R*_PA_ for pimelic acid content. Citric acid and the relevant diacid were mixed as solids in a glass vial before heating (hot plate) until a homogeneous liquid was obtained; a slow heating rate was required to avoid bubble formation. The combined hardeners were then cooled to just above their crystallization temperature, where ELO (or GTE), preheated to the same temperature, was added and mixed with a magnetic stirrer until the mixture appeared homogeneous, typically in less than a minute. The mixing temperatures varied from 100–105 °C. Epoxy thermosets with no citric acid were synthesized by mixing ELO and diacid in a vial with a magnetic stirrer and heating until a homogeneous mixture was obtained, typically just below the diacid melting point. Preresin mixtures were poured into silicone molds preheated to 100 °C on a hot plate and cured in a convection oven at 160 °C for 2 h.

## Results and Discussion

Preliminary investigations were conducted using a range of linear aliphatic diacids such as succinic acid, adipic acid, glutaric acid (GA), pimelic acid (PA), and azelaic acid. Succinic acid was not readily homogenized with ELO at temperatures low enough to prevent immediate gelation, while longer chain diacids (such as azelaic acid) were not chosen for this study due to the anticipated reduction in rigidity of the resulting thermoset. Based on the observation that the diacids GA and PA could homogenize citric acid (CA) and ELO at low temperature, while also being suitable, bio-based, cheap cross-linkers, it was decided to investigate the solvent-free formation of epoxy thermosets using these widely available precursors. Toward this, several series of epoxy thermosets were synthesized using CA, ELO, and one of the two chosen diacid hardeners, variously GA or PA. The ratio of carboxylic acid groups to epoxide groups is the key variable and will herein be referred to as *R*_tot_ as described above and outlined in eq 1. *R*_tot_ is also represented as the sum of the acid to epoxide ratios for the constituent acids as per eq 2.

1 $$R_{tot}=\left[COOH\right]/\left[epoxide\right]$$

2 $$R_{tot}=R_{CA}+R_{GA/PA}$$

*R*_tot_ was varied to establish epoxy thermoset formation for all ratios from 0.6 to 0.8. Within this range, for various values of *R*_tot_, the ratio between the molar quantity of the trifunctional citric acid-derived acid moieties (*R*_CA_) and those associated with difunctional GA (*R*_GA_) or PA (*R*_PA_) was varied, and any resulting changes in the observed properties of the thermosets were recorded. For example, an ELO thermoset with *R*_tot_ = 0.8 could consist of *R*_CA_ 0.6 and *R*_GA_ 0.2, where because there are 5.5 epoxide groups on average in ELO, eq 1 would lead to 0.8 = [COOH]/5.5 resulting in a [COOH] of 5.5 × 0.8 = 4.4. This leads to 4.4 carboxy group equivalents per 5.5 epoxide group equivalents with 3.3 carboxy groups coming from the CA and 1.1 coming from the relevant diacid hardener.

All liquid formulations shown in this study were visually homogeneous prior to curing. All of the epoxy thermosets were cured at 160 °C for 2 h, which resulted in hard, clear, bubble-free, and visibly homogeneous materials. Curing at lower temperatures was attempted but consistently led to softer materials, presumably due to incomplete cross-linking. As *R*_CA_ increased relative to *R*_GA/PA_ the synthesis of the thermosets became more challenging due to an increase in viscosity, with the reaction proceeding too rapidly for molding. The critical value for successful synthesis of GA thermosets was an *R*_tot_/*R*_CA_ of 0.69 to 0.82 (Table S1), while PA allowed for an increase in citric acid content to *R*_tot_/*R*_CA_ of 0.75 to 0.90.

### Mechanical Properties

Mechanical performance under tensile stress is a key indicator of the strength and durability of a resin/thermoset material. Accordingly, mechanical (tensile) testing was performed on the GA-ELO thermosets to gain insight into their properties. Thermoset samples for tensile testing were cured in a dog-bone-shaped mold, yielding the appropriate shape and dimensions for analysis. Samples were then placed between the clamps of the test apparatus, permitting determination of their tensile strength, *E* and elongation at break. Initially, testing focused on the CA/GA thermosets prepared with ELO. Each thermoset had been cured in a mold of a Type 1BA dog bone to ensure consistent and uniform sample dimensions. Mechanical properties were assessed for thermosets of each *R*_tot_ value and acid composition for which a homogeneous thermoset could be successfully prepared. Stress–strain curves were then used to derive tensile strength (MPa), Young’s modulus (*E*, MPa), toughness (J/m^3^), and elongation at break (%, Figure 1).

**Figure 1:**
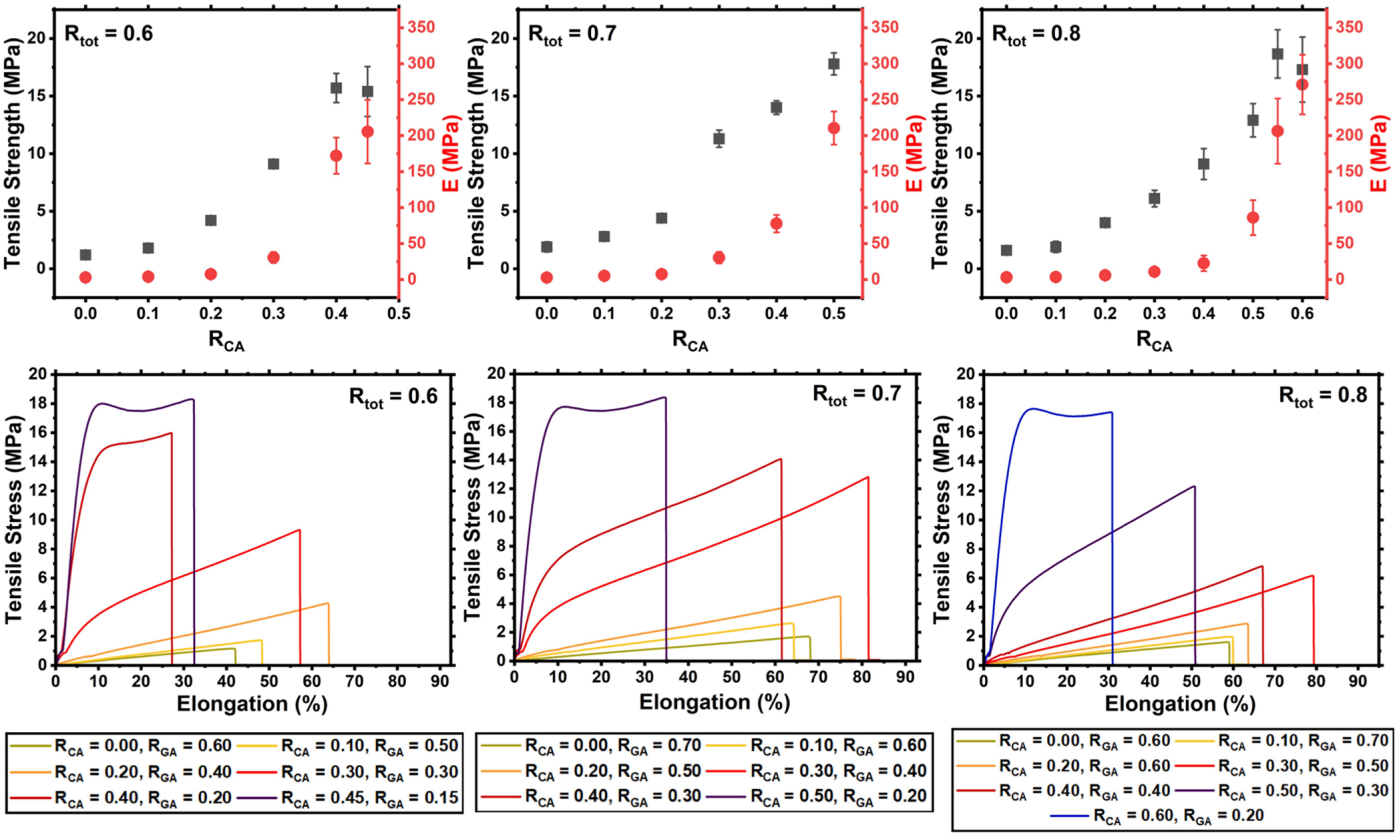
Top panel: Tensile strength vs *E* values vs *R*_CA_ for the ELO/GA epoxy thermosets with *R*_tot_ = 0.6 (L), 0.7 (center) or 0.8 (R) with varying values of *R*_CA_ and *R*_GA_. Bottom panel: Tensile stress (MPa) vs elongation (%) of epoxy thermosets made from ELO, CA, and GA with *R*_tot_ = 0.6 (L), 0.7 (center), or 0.8 (R) with varying values of *R*_CA_ and *R*_GA_.

For all values of *R*_tot_, both the tensile strength and *E* increased with the *R*_CA_ value. This is consistent with a higher cross-linking density in high-CA systems. Moreover, where *R*_tot_ was highest (0.8), it was possible to prepare a homogeneous preresin containing a higher absolute concentration of CA than is achievable at lower *R*_tot_ values, with the resulting material exhibiting a higher *E* value. Notably, however, tensile strength increased more rapidly with increasing *R*_CA_ in the systems where *R*_tot_ = 0.6 and *R*_tot_ = 0.7, relative to those where *R*_tot_ = 0.8, allowing materials with relatively high strengths to be readily accessed from preresin formulations that are sufficiently slow curing to facilitate ease of handling in a process setting.

For all GA systems assessed, elongation at break was highest for materials with intermediate values of *R*_CA_, with both high- and low-*R*_CA_ systems breaking at comparatively lower elongations, consistent with a moderately high cross-linking density and retention of some elasticity (Figure 1). It is, nonetheless, plausible that this trend may be partially attributable to the increased viscosity and rapid curing of high-CA (and generally high-acid) formulations, limiting the extent to which the epoxide monomer and hardener are able to react. Similarly, the greater tensile strength of the thermosets for which *R*_CA_ = 0.3 and *R*_CA_ = 0.4, where *R*_tot_ = 0.6 and *R*_tot_ = 0.7, relative to systems of otherwise comparable composition where *R*_tot_ = 0.8, may find a similar basis. For comparison, mechanical testing was also performed for all *R*_tot_ values of the CA/PA thermosets prepared with ELO using the same analysis method. The trends corresponding to each *R*_tot_ value are shown in Figure 2.

**Figure 2:**
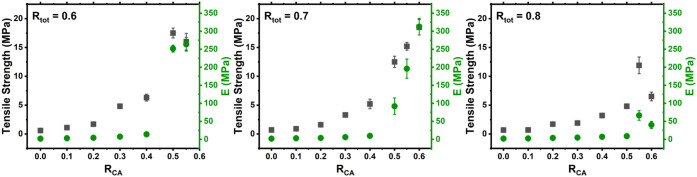
Tensile strength vs *E* values vs *R*_CA_ for the ELO/PA epoxy thermosets with varying *R*_tot_ values.

As with the CA/GA/ELO thermosets, their PA analogues also showed increased tensile strength and *E* as the *R*_CA_ increased. However, at *R*_tot_ = 0.8, the tensile strength and *E* were greatly reduced compared with the other *R*_tot_ values. Additionally, all of the PA thermosets showed marginally reduced mechanical performance (aside from the *R*_tot_ = 0.8 thermosets) when compared with their GA analogues, potentially due to the longer carbon chain present in PA resulting in a slightly lower cross-linking density within the thermoset.

### Thermal Properties

Having assessed the various thermoset formulations’ mechanical performance, we next assessed the thermal properties of the CA/GA and CA/PA thermosets using thermogravimetric analysis (TGA) and differential scanning calorimetry (DSC). It was hypothesized that the stronger high-*R*_CA_ thermosets would exhibit higher *T*_g_ values, indicative of increased cross-linking density.^55^ The entire series of synthesized thermosets with *R*_tot_ = 0.6 – 0.8 were analyzed by DSC to obtain *T*_g_ values (an example is shown in Figure 3). Additionally, DSC was conducted to investigate how the crystallization temperature of the mixture relates to its molar composition (Figure S1) with no discernible crystallization peak observed. The first heating cycle of the DSC was used for determination of *T*_g_ as it most accurately reflected the properties of the produced materials. It should be noted that higher temperature events, above the *T*_g_, could be observed in the DSC > 100 °C for all thermosets, this being attributed to further curing events occurring, due to the presence of residual unreacted acid and epoxide moieties. Despite this, only small increases in *T*_g_ are observed in the second heating scan, for example, from 6.6 to 10.9 °C for *R*_GA_/*R*_tot_ = 0.6 (Figure S2). In both the GA and PA series, *T*_g_ values varied from −5 to 30 °C, with strong correlations for increased CA content and higher *T*_g_. In addition, increased total acid content (*R*_tot_) correlated with increased *T*_g_ when comparing the similar ratios of CA:GA/PA (Figure 3), consistent with the ratio of acid and epoxide groups approaching a stoichiometric value. When compared to mechanical data, *T*_g_ and associated increases in *R*_GA_ or *R*_PA_ correlate with increased toughness, strength, *E*, and a decrease in elongation (Figures 1, 2, and 3). Dynamic Mechanical Analysis (DMA) was carried out on several thermosets from the *R*_tot_ = 0.6 series. As expected from DMA,^55,56^ the measured *T*_g_ values, taken from *E″* maxima, are slightly raised compared to DSC *T*_g_ (∼10–15 °C); however, the series shows the same clear trend of *T*_g_ with *R*_CA_:*R*_GA_ (Figures S3 and S4).

**Figure 3:**
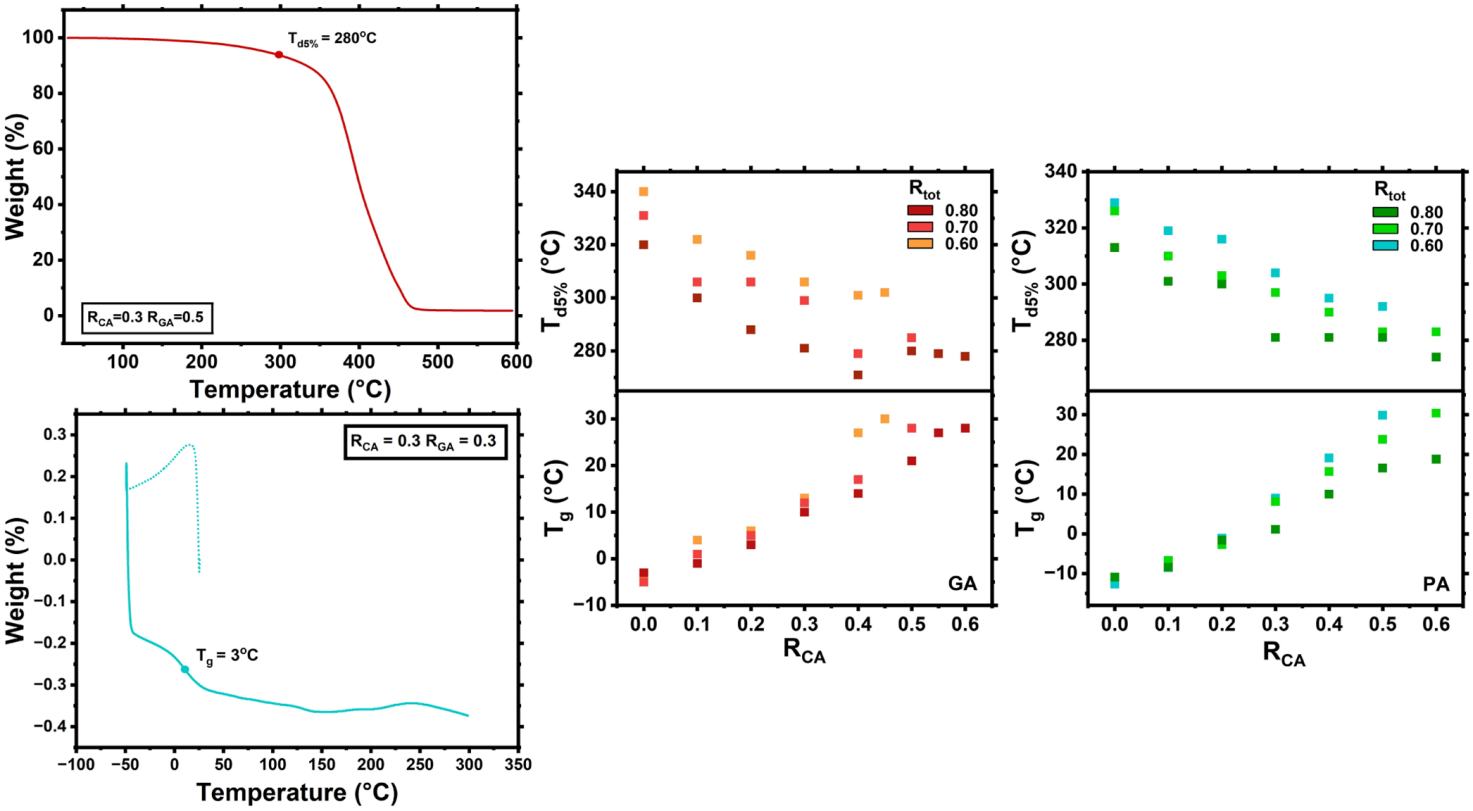
Example TGA (L—top) and DSC (L—bottom) of selected *R*_tot_ = 0.8 and *R*_tot_ = 0.6 CA/GA thermosets. Temperature of 5 wt % degradation measured by TGA (R—top) and *T*_g_ measured by DSC (R—bottom) of thermosets vs *R*_CA_ and *R*_tot_ for the epoxy thermosets made from ELO, CA, and GA (orange—red) and PA (teal—green).

With the observed strong indication that increased cross-linking affords higher *T*_g_ and thermoset strength, TGA was undertaken on the thermosets to assess their high-temperature stability and decomposition profiles. All thermosets exhibited a single-step sigmoidal decomposition profile, consistent with the uniformity in their curing chemistry. The onset of degradation is linked to the thermal stability of a material and is typically taken at 5 wt % mass loss (*T*_d5%_).^57^ The *T*_d5%_ was recorded for all thermosets, with values from 271 to 340 °C. A clear trend was observed, with higher *R*_tot_ and *R*_CA_ or *R*_PA_ correlating with lower degradation onset (Figure 3). All TGA thermograms are shown in Figures S5–S 10. It is hypothesized that increased content of unreacted chain-end acid groups, in the higher *R*_tot_ thermosets, may plausibly lead to a slight increase in acid-catalyzed degradation, and therefore a drop in *T*_d5%_. For all thermosets, <5 wt % solids remained after the temperature exceeded 500 °C, which is consistent with minimal carbonization upon pyrolysis.

### Degradation and Swelling Properties

The swelling index (%, toluene) was determined for each thermoset to gain an understanding of the cross-link density resulting in the case of each composition. Additionally, the gel content (%, toluene) of each thermoset was measured so that the amount of acid incorporated into the thermoset could be determined. The gel content for each thermoset was high, >93% for PA-based systems and >96% where GA was used, characteristic of thermoset formation (Figure S11). The swelling index values for GA thermosets show that at all values of *R*_GA_, the swelling index decreases as the *R*_CA_ value increases (Figure 4). Another observation is that overall, the swelling index decreases as *R*_CA_ increases. The results from the swelling of the PA thermosets show that for all values of *R*_PA_, the swelling index of the thermoset decreases as you increase the *R*_CA_ value. This change is particularly evident for PA samples that have *R*_tot_ = 0.8 as the swelling index decreases from 73% with no CA present to 37% when an *R*_CA_ value of 0.55 is present. Additionally, the PA thermosets swell more than their GA analogues, possibly due to their higher carbon chain length compared with GA. Water absorption analysis showed minimal water absorption (<2.5%) over 168 h for all thermosets.

**Figure 4:**
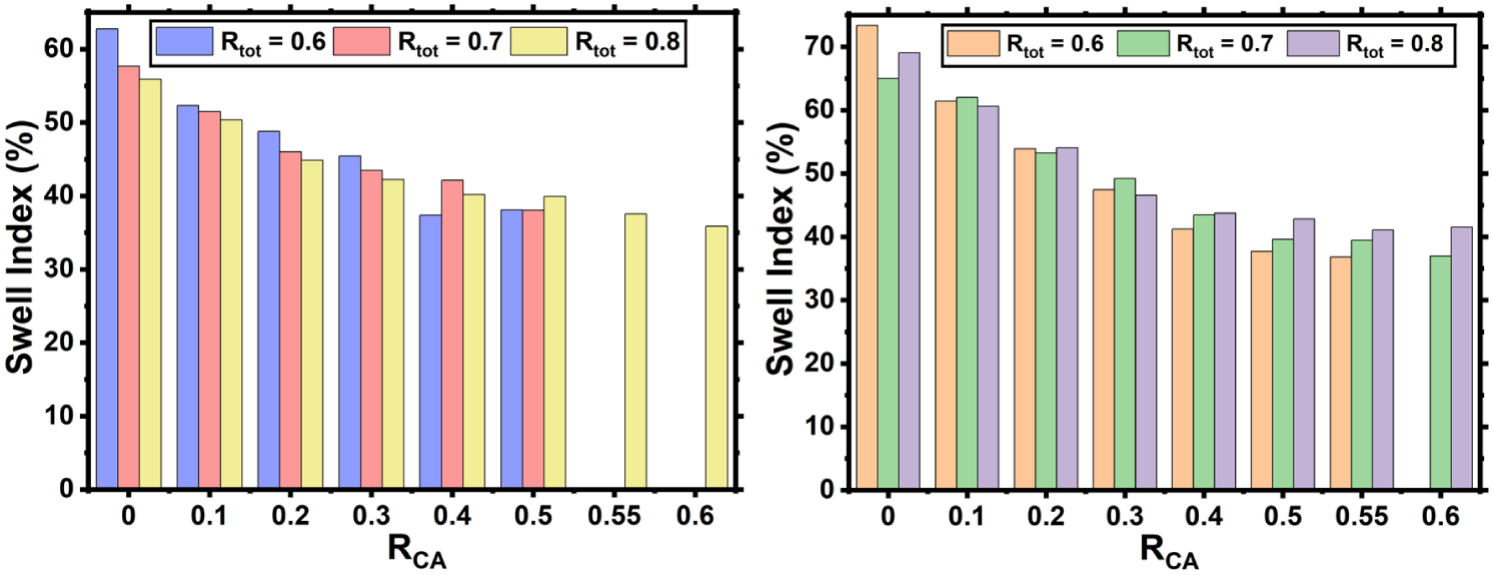
Swelling index (%, in toluene) of the epoxy thermosets prepared from either GA (L) or PA (R) and varying *R*_CA_ amounts at different *R*_tot_ values.

To ascertain whether the thermosets are degradable or not, both series of *R*_tot_ = 0.7 GA thermosets were exposed to alkaline (1 M NaOH) degradation conditions over a period of 24 h (at 60 °C) or 168 h (at room temperature). Additionally, the thermosets were also left exposed to aqueous conditions over 168 h with no degradation being observed. The degradation profiles obtained from the alkaline degradation experiments are shown in Figure 5 and Figure S12.

**Figure 5:**
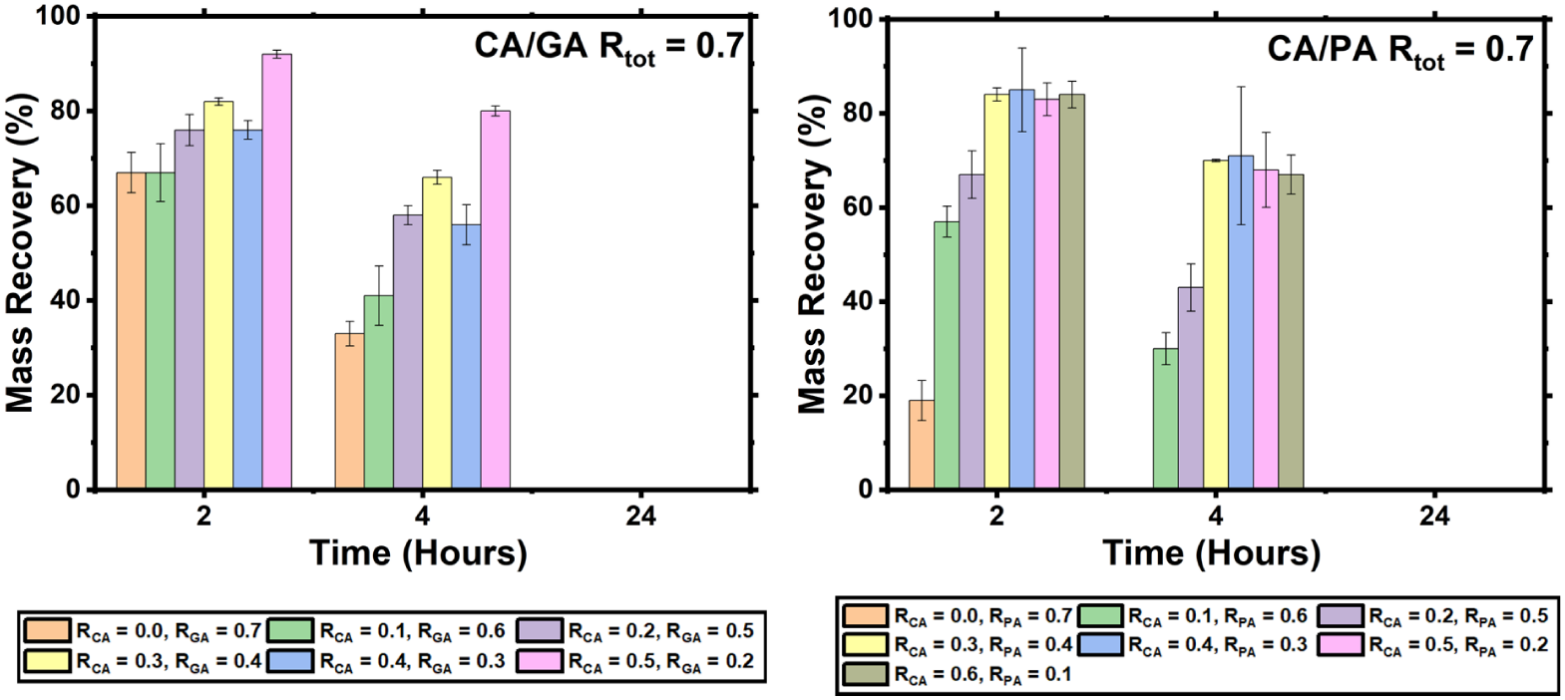
Mass recovery (%) of the epoxy thermosets (*R*_tot_ = 0.7) prepared from CA/GA (L) and CA/PA (R) after degradation when exposed to 1 M NaOH at 60 °C. All samples displayed full degradation after 24 h.

When exposed to an alkaline solution at room temperature, the thermosets showed a tendency to degrade with samples of thermosets with an *R*_CA_ value of 0.0 – 0.3 fully degrading over 120 h (Figure S12). The sample with an *R*_CA_ value of 0.5 had not fully degraded by 168 h but had shown a mass loss of 83%. Contrastingly, when the same thermosets are exposed to 1 M NaOH at 60 °C, they all fully degraded within 24 h. The series of *R*_tot_ = 0.7 PA thermosets was also exposed to a 1 M NaOH solution at 60 °C to follow their degradation profile, with their results being shown in Figure 5. All samples displayed complete degradation into solution after 24 h with the sample that did not contain CA degrading the fastest (4 h). This contrasts with the analogous sample from the GA series, which only fully degraded after 24 h.

These encouraging results show that both ELO/GA and ELO/PA epoxy thermosets are degradable and can be broken down under relatively facile and gentle alkaline conditions over 24 h, further adding to their suitability for sustainable applications.

### Performance Testing with a Different Epoxide Cross-Linker

While results with ELO thermosets are very promising for a range of applications, bio-based materials with higher maximum tensile strength are needed as alternatives to high strength materials like widely used bisphenol epoxy thermosets.^58,59^ Toward this goal, the shorter chain trifunctional epoxide, glycerol triglycidyl ether (GTE), with a high potential for biosourcing,^60^ was investigated in the CA/GA system. Two thermoset formulations were made with an *R*_tot_ of 0.6 (*R*_GA_ and *R*_CA_ = 0.3) and 0.7 (*R*_CA_ = 0.5, *R*_GA_ = 0.2), both proceeding *via* a homogeneous preresin, similar to the ELO-based systems. Upon formation, it was immediately noted that the GTE-based thermoset in which *R*_tot_ = 0.7, with high CA content, was tougher and stiffer than the thermosets previously created using ELO. The two GTE-based thermosets were therefore subjected to mechanical testing, with their properties exhibiting stark differences relative to the ELO-based thermosets (Figure 6). The thermoset with *R*_tot_ = 0.6 afforded an elongation of over 70%, exceeding all ELO *R*_tot_ = 0.6 thermosets, with a maximum tensile strength also exceeding those of the respective ELO analogues. In contrast, the *R*_tot_ = 0.7 GTE thermoset permitted minimal elongation and exhibited a maximum tensile strength of over 48 MPa, more than double the strength of any of the ELO thermosets. Such remarkable strength observed in these preliminary investigations into GTE-based materials is comparable to acrylonitrile-butadiene-styrene (ABS) plastic^61^ and some bisphenol epoxy thermosets.^58,59^

**Figure 6:**
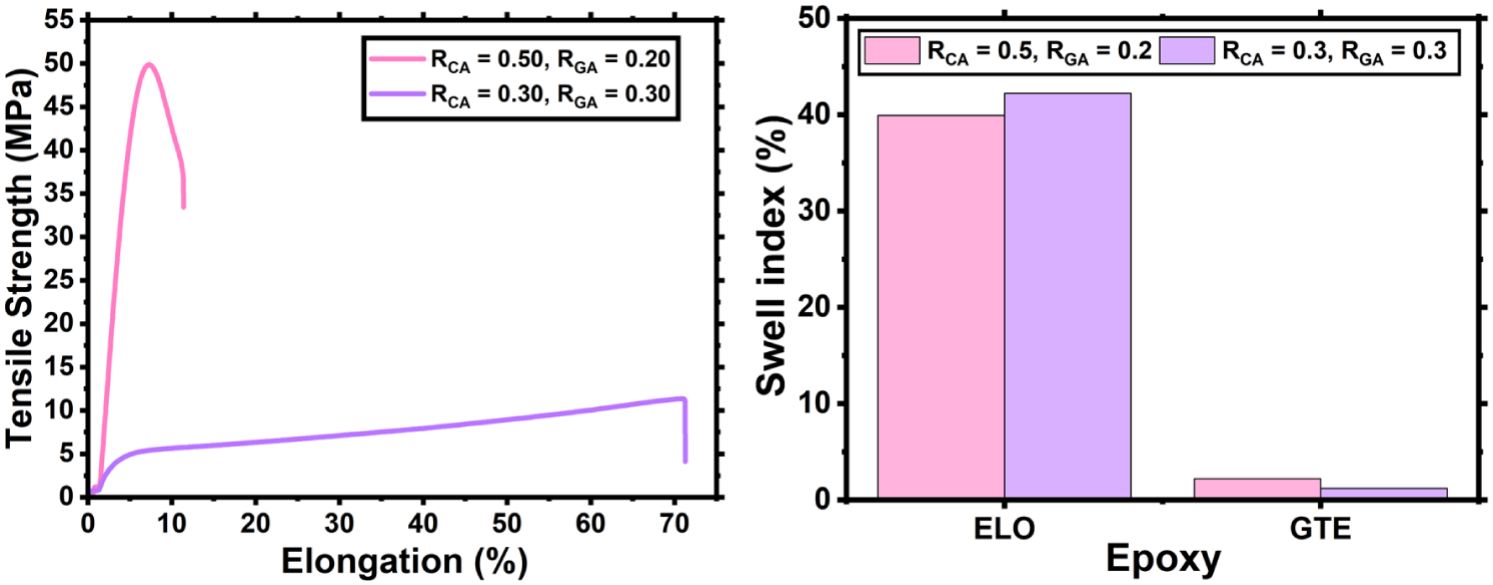
Comparison of the tensile strength vs elongation of epoxy thermosets made from GTE, CA, and GA with varying values of *R*_tot_, *R*_CA_, and R_GA_ (L) and the swelling index (%, in toluene) and of the epoxy thermosets prepared from GA and CA at different *R*_tot_ values with either ELO or GTE as the epoxy (R).

It is hypothesized that the short-chain nature of glycerol triglycidyl ether (GTE), compared to ELO, results in a greater cross-linking density and therefore increased strength. To corroborate this, a toluene swelling test was conducted on the CA/GA epoxy thermosets with either ELO or GTE present (Figure 6). The swell index was drastically reduced when using GTE instead of ELO, from roughly 40% with ELO present to < 4% with GTE present, consistent with the hypothesis of a high cross-link density. Thus, thermosets made with GTE rather than ELO yield a more rigid, higher density gel matrix with less swell, less flexibility, but drastically higher strength. These preliminary results show the potential beneficial properties that the epoxy thermosets can possess if GTE is used as the epoxy source when compared with ELO. Future studies could look at the effect of cross-link density on the thermomechanical properties of thermosets prepared with GTE as well as their likelihood to degrade. Moreover, it is conceivable that preparation of thermosets containing mixed epoxide monomers, comprising both ELO and GTE resins in various ratios, may afford a suite of materials exhibiting a continuum of properties intermediate between those of the purely ELO- and GTE-based systems.

## Conclusion

We have reported the solvent-free, catalyst-free, and additive-free synthesis of fully bio-based epoxy thermosets consisting of multifunctional aliphatic acids and epoxides obtained from vegetable oils. The formulation was optimized through controlled variation of the ratio of liquid diacid to (trifunctional) citric acid, facilitating homogenization of the latter, solid and highly polar acid with the chosen diacid hardener at moderate temperature. Mechanical properties were found to be tunable, being strongly associated with thermoset composition, with the materials’ ultimate tensile strength ranging from below 1 to 20 MPa and elongation at break varying from less than 30% to over 80%. Promisingly for end-of-life considerations, accelerated degradation under alkaline conditions resulted in complete degradation of all thermosets in under 24 h. By replacing the ELO epoxide source with GTE, preliminary results indicate that the resulting thermosets have further, dramatically, enhanced strength while also showcasing the generality of our thermoset preparation method. While these systems offer a green alternative for less demanding applications, they do not yet match the performance of conventional epoxy resins. Nevertheless, the tunability of GTE and short-chain diacids provides a clear route to better-performing bio-based thermosets.

## Supplementary Material



## Notes

Experimental details, additional DSC thermograms, DMA plots, TGA thermograms, gel content information, and additional degradation profiles (PDF)

## References

[1] Jiang Y., Li J., Li D., Ma Y., Zhou S., Wang Y., Zhang D. (2024). Bio-Based Hyperbranched Epoxy Resins: Synthesis and Recycling. Chem. Soc. Rev..

[2] Feng Y., Zhang Z., Yue D., Belko V. O., Maksimenko S. A., Deng J., Sun Y., Yang Z., Fu Q., Liu B., Chen Q. (2024). Recent Progress
in Degradation and Recycling of Epoxy
Resin. J. Mater. Res. Technol..

[3] Liang M., Liu X., Liu D., Li X., Hu X., Feng C., Li T.-T., Lin J.-H., Chang B., Chen J. (2024). A Review of
the Curing Rate and Mechanical Properties of Epoxy Resin on Polymer
Matrix Composites. J. Polym. Res..

[4] Liu S., Liu Z., Jiang D., Zong R., Zhong A., Shentu B. (2025). Synergistic
Effects of Tertiary Amine and Imidazole Accelerators on Epoxy Resin
Curing. J. Appl. Polym. Sci..

[5] Mu M., Vaughan A. (2021). Dielectric Behaviours
of Bio-Derived Epoxy Resins from
Cashew Nutshell Liquid. High Voltage.

[6] Yu K., Shi Q., Dunn M. L., Wang T., Qi H. J. (2016). Carbon Fiber Reinforced
Thermoset Composite with Near 100% Recyclability. Adv. Funct. Mater..

[7] Reddy K. S. K., Gao W.-J., Chen C.-H., Juang T.-Y., Abu-Omar M. M., Lin C.-H. (2021). Degradation of Thermal-Mechanically
Stable Epoxy Thermosets,
Recycling of Carbon Fiber, and Reapplication of the Degraded Products. ACS Sustainable Chem. Eng..

[8] Klose L., Meyer-Heydecke N., Wongwattanarat S., Chow J., Pérez
García P., Carré C., Streit W., Antranikian G., Romero A. M., Liese A. (2023). Towards Sustainable Recycling of
Epoxy-Based Polymers: Approaches and Challenges of Epoxy Biodegradation. Polymers.

[9] Almushaikeh A. M., Alaswad S. O., Alsuhybani M. S., AlOtaibi B. M., Alarifi I. M., Alqahtani N. B., Aldosari S. M., Alsaleh S. S., Haidyrah A. S., Alolyan A. A., Alshammari B. A. (2023). Manufacturing of Carbon Fiber Reinforced
Thermoplastics and Its Recovery of Carbon Fiber: A Review. Polym. Test..

[10] De B., Bera M., Bhattacharjee D., Ray B. C., Mukherjee S. (2024). A Comprehensive
Review on Fiber-Reinforced Polymer Composites: Raw Materials to Applications,
Recycling, and Waste Management. Prog. Mater.
Sci..

[11] Vidil T., Tournilhac F., Musso S., Robisson A., Leibler L. (2016). Control of
Reactions and Network Structures of Epoxy Thermosets. Prog. Polym. Sci..

[12] Liu Y.-Y., Liu G.-L., Li Y.-D., Weng Y., Zeng J.-B. (2021). Bio-based
High-Performance Epoxy Vitrimer with UV Shielding for Recyclable Carbon
Fiber Reinforced Composites. ACS Sustainable
Chem. Eng..

[13] Kavalli T., Wolf R., Ozen S., Lalevée J. (2022). Comparison
of Pure Epoxy vs. Epoxy-Anhydride Photopolymerization. Eur. Polym. J..

[14] Lerma-Canto A., Samper M. D., Dominguez-Candela I., Garcia-Garcia D., Fombuena V. (2023). Epoxidized and Maleinized Hemp Oil to Develop Fully
Bio-Based Epoxy Resin Based on Anhydride Hardeners. Polymers.

[15] Yamamoto S., Ida R., Aoki M., Kuwahara R., Shundo A., Tanaka K. (2023). Formation
Mechanism of a Heterogeneous Network in Epoxy Resins. Macromolecules.

[16] Pal S., Dansuk K., Giuntoli A., Sirk T. W., Keten S. (2023). Predicting
the Effect of Hardener Composition on the Mechanical and Fracture
Properties of Epoxy Resins Using Molecular Modeling. Macromolecules.

[17] Dağlar Ö., Eisenreich F., Tomović Ž. (2024). Chemical and Solvent-Based Recycling
of High-Performance Epoxy Thermoset Utilizing Imine-Containing Secondary
Amine Hardener. Adv. Funct. Mater..

[18] Varganici C.-D., Rosu L., Rosu D., Rosca I., Ignat M.-E., Ignat L. (2024). Surface Degradation
of DGEBA Epoxy Resins Cured with Structurally
Different Amine Hardeners: Effects of UV Radiation. Polymers.

[19] Sheng Q., Chen Q., Gu W., Wang R., Gu X., Liu J., Sun T., Chen Y., Sun J., Zhang S. (2024). The Study
of Curing Behavior and Thermo-Mechanical Properties of Epoxy Adhesives
with Different Anhydrides. Polymer.

[20] Aslanova E. T., Heydarova S. Y., Iskenderova E. G., Mamedov B. A. (2024). New Epoxy-Imide
Resin. Inorg. Mater. Appl. Res..

[21] Jaillet F., Desroches M., Auvergne R., Boutevin B., Caillol S. (2013). New Bio-based
Carboxylic Acid Hardeners for Epoxy Resins. Eur. J. Lipid Sci. Technol..

[22] Ozeren
Ozgul E., Ozkul M. H. (2018). Effects of Epoxy, Hardener, and Diluent
Types on the Workability of Epoxy Mixtures. Constr. Build. Mater..

[23] Ozeren
Ozgul E., Ozkul M. H. (2018). Effects of Epoxy, Hardener, and Diluent
Types on the Hardened State Properties of Epoxy Mortars. Constr. Build. Mater..

[24] Hevus I., Ricapito N. G., Tymoshenko S., Raja S. N., Webster D. C. (2020). Bio-based
Carboxylic Acids as Components of Sustainable and High-Performance
Coating Systems. ACS Sustainable Chem. Eng..

[25] Fantoni A., Koch T., Baudis S., Liska R. (2023). Synthesis and Characterization
of Homogeneous Epoxy Networks: Development of a Sustainable Material
Platform Using Epoxy-Alcohol Polyaddition. ACS
Appl. Polym. Mater..

[26] Nepomuceno N. C., Barreto V., Wellen R. M. R. (2024). Effect of Dicarboxylic Acids’
Aliphatic Chain on the Curing of Epoxidized Soybean Oil (ESO) Resins. J. Polym. Environ..

[27] Kim D., Yu C., Kwon Y., Kim J., Chung K., Kim H.-J., Kwon M. S. (2023). Correlation between
the Structural Variations in Thiol-Based
Hardeners and Properties of Thiol–Epoxy Polymers. ACS Appl. Polym. Mater..

[28] Kim Y.-H., Baek J. J., Chang K. C., Park B. S., Koh W.-G., Shin G. (2023). Effect of Synthetic
Low-Odor Thiol-Based Hardeners Containing Hydroxyl
and Methyl Groups on the Curing Behavior, Thermal, and Mechanical
Properties of Epoxy Resins. Polymers.

[29] Song J. H., Hong S.-M., Park S. K., Kwon H. K., Hwang S.-H., Oh J.-M., Koo S.-M., Lee G., Park C. (2025). Synthesis
and Characterization of Hyperbranched Thiol Hardener and Their Curing
Behavior in Thiol–Epoxy. Macromol. Res..

[30] Kim G., Song I. W., Jang J., Hwang J., Nam K., Kwon M. S. (2025). Enhancing
Epoxy Toughness with a Bis-Hydroxy Urethane
Diol Additive Derived from CO2-Based Ethylene Carbonate and Bio-Based
Diamine. ACS Appl. Eng. Mater..

[31] Galego N., Vázquez A., Williams R. J. J. (1994). The First Stages of an Epoxy Homopolymerization
Initiated by Piperidine. Polymer.

[32] Edinger D., Fischer S. M., Slugovc C. (2024). Functionalized
Electron-Rich Pyridines
as Initiators for the Epoxy Homopolymerization. Macromol. Chem. Phys..

[33] Lavaux V., Lalevée J. (2024). Epoxy Curing in Mild and Eco-Friendly Conditions: Towards
Bisphenol A-Free Systems. Prog. Polym. Sci..

[34] Chen J.-S., Ober C. K., Poliks M. D., Zhang Y., Wiesner U., Cohen C. (2004). Controlled Degradation
of Epoxy Networks: Analysis of Crosslink Density
and Glass Transition Temperature Changes in Thermally Reworkable Thermosets. Polymer.

[35] Kim B., Choi J., Yang S., Yu S., Cho M. (2015). Influence
of Crosslink Density on the Interfacial Characteristics of Epoxy Nanocomposites. Polymer.

[36] Huang J., Fu P., Li W., Xiao L., Chen J., Nie X. (2022). Influence
of Crosslinking Density on the Mechanical and Thermal Properties of
Plant Oil-Based Epoxy Resin. RSC Adv..

[37] Orselly M., Devemy J., Bouvet-Marchand A., Dequidt A., Loubat C., Malfreyt P. (2022). Molecular Simulations
of Thermomechanical Properties
of Epoxy-Amine Resins. ACS Omega.

[38] Huang K., Zhang P., Zhang J., Li S., Li M., Xia J., Zhou Y. (2013). Preparation of Bio-based Epoxies Using Tung Oil Fatty
Acid-Derived C21 Diacid and C22 Triacid and Study of Epoxy Properties. Green Chem..

[39] Di
Mauro C., Malburet S., Genua A., Graillot A., Mija A. (2020). Sustainable Series of New Epoxidized Vegetable Oil-Based Thermosets
with Chemical Recycling Properties. Biomacromolecules.

[40] Malburet S., Di Mauro C., Noè C., Mija A., Sangermano M., Graillot A. (2020). Sustainable Access to Fully Bio-based Epoxidized Vegetable
Oil Thermoset Materials Prepared by Thermal or UV-Cationic Processes. RSC Adv..

[41] Álvarez M., Reilly A., Suleyman O., Griffin C. (2025). A Systematic Review
of Epoxidation Methods and Mechanical Properties of Sustainable Bio-Based
Epoxy Resins. Polymers.

[42] Altuna F. I., Pettarin V., Williams R. J. J. (2013). Self-Healable
Polymer Networks Based
on the Cross-Linking of Epoxidised Soybean Oil by an Aqueous Citric
Acid Solution. Green Chem..

[43] Kadam A., Pawar M., Yemul O., Thamke V., Kodam K. (2015). Biodegradable
Bio-based Epoxy Resin from Karanja Oil. Polymer.

[44] Nepomuceno N. C., Bakkali-Hassani C., Wellen R., Caillol S., Negrell C. (2024). Fully Bio-Sourced
Catalyst-Free Covalent Adaptable Networks from Epoxidized Soybean
Oil and L-Tartaric Acid. Eur. Polym. J..

[45] Chong K. L., Lai J. C., Abd Rahman R., Adrus N., Al-Saffar Z. H. (2021). Self-Healable
Bio-Based Epoxy Resin from Epoxidized Palm Oil. Chem. Eng. Trans..

[46] Tellers J., Willems P., Tjeerdsma B., Guigo N., Sbirrazzuoli N. (2020). Eutectic Hardener
from Food-Based Chemicals to Obtain Fully Bio-Based and Durable Thermosets. Green Chem..

[47] Necolau M. I., Damian C. M., Olaret E., Iovu H., Balanuca B. (2022). Comparative
Thermo-Mechanical Properties of Sustainable Epoxy Polymer Networks
Derived from Linseed Oil. Polymers.

[48] Tellers J., Jamali M., Willems P., Tjeerdsma B., Sbirrazzuoli N., Guigo N. (2021). Cross-Linking Behavior
of Eutectic
Hardeners from Natural Acid Mixtures. Green
Chem..

[49] Ding C., Shuttleworth P. S., Makin S., Clark J. H., Matharu A. S. (2015). New Insights
into the Curing of Epoxidized Linseed Oil with Dicarboxylic Acids. Green Chem..

[50] Sahoo S. K., Khandelwal V., Manik G. (2018). Development of Completely Bio-Based
Epoxy Networks Derived from Epoxidized Linseed and Castor Oil Cured
with Citric Acid. Polym. Adv. Technol..

[51] Hood C., Ghazani S. M., Marangoni A. G., Pensini E. (2022). Flexible Polymeric
Biomaterials from Epoxidized Soybean Oil, Epoxidized Oleic Acid, and
Citric Acid as Both a Hardener and Acid Catalyst. J. Appl. Polym. Sci..

[52] Moser B. R., Cermak S. C., Evangelista R. L. (2024). Fully Bio-based
Epoxy Resins from
Ring Opening Polymerization of Epoxidized Pennycress (*Thlaspi
Arvense* L.) Oil with Itaconic and Citric Acids. Ind. Crops Prod..

[53] Jamali-Moghadam-Siahkali M., Guigo N., Vincent L., Sbirrazzuoli N. (2024). Low-Viscosity
Eutectic Hardeners for More Sustainable Cross-Linking Conditions. ACS Appl. Polym. Mater..

[54] Anusic A., Blößl Y., Oreski G., Resch-Fauster K. (2020). High-Performance
Thermoset with 100% Bio-Based Carbon Content. Polym. Degrad. Stab..

[55] Bandzierz K., Reuvekamp L., Dryzek J., Dierkes W., Blume A., Bielinski D. (2016). Influence of Network Structure on Glass Transition
Temperature of Elastomers. Materials.

[56] Wang R., Schuman T. P. (2013). Vegetable Oil-Derived
Epoxy Monomers and Polymer Blends:
A Comparative Study with Review. Express Polym.
Lett..

[57] Gu A., Liang G. (2003). Thermal Degradation
Behaviour and Kinetic Analysis of Epoxy/Montmorillonite
Nanocomposites. Polym. Degrad. Stab..

[58] Liu H., Wu X., Liu Y., Guo Z., Ge Q., Sun Z. (2022). The Curing
Characteristics and Properties of Bisphenol A Epoxy Resin/Maleopimaric
Acid Curing System. J. Mater. Res. Technol..

[59] Gu H., Cao Q., Li J., Zhao J., Zhang S., Jian X., Weng Z. (2023). Enhancing
the Comprehensive Performance of Bisphenol A Epoxy Resin
via Blending with a Bio-Based Counterpart. Polymer.

[60] Lari G. M., Pastore G., Mondelli C., Pérez-Ramírez J. (2018). Towards Sustainable
Manufacture of Epichlorohydrin from Glycerol Using Hydrotalcite-Derived
Basic Oxides. Green Chem..

[61] Olivera S., Muralidhara H. B., Venkatesh K., Gopalakrishna K., Vivek C. S. (2016). Plating on Acrylonitrile–Butadiene–Styrene
(ABS) Plastic: A Review. J. Mater. Sci..

