# Supplementary material for: Bio-Based, Degradable, and Tunable Epoxy Thermosets from Homogenized Acids and Epoxidized Linseed Oil or Glycerol Triglycidyl Ether
Source: ACS Sustain Chem Eng. 2025 Dec 17;14(1):586–94. doi: 10.1021/acssuschemeng.5c10683 (PMC12801383; doi:10.1021/acssuschemeng.5c10683)
Supplement: Supplementary file 1 [file sc5c10683_si_001.pdf]

Supporting Information

Bio-Based, Degradable and Tunable Epoxy Thermosets from Homogenized Acids and Epoxidized Linseed Oil or Glycerol Triglycidyl Ether.

Benjamin J. Groombridge, Gavin R. Irvine*, Vlad Jarkov, Jonathan T. Husband, Strachan N. McCormick, Matthew G. Davidson*.

Institute of Sustainability and Climate Change and Department of Chemistry, University of Bath, Bath, BA2 7AY, United Kingdom

***Corresponding authors:** Gavin R. Irvine and Matthew G. Davidson.

E-mail: gi217@bath.ac.uk, chsmgd@bath.ac.uk

Number of pages: 9

Number of figures: 12

Number of tables: 1

Materials

Epoxidized Linseed Oil (ELO) was kindly provided by Valtris Chemicals (Manchester, UK, molecular weight of 980 g mol^{-1}, an average of 5.5 epoxy groups per molecule and an epoxy equivalent weight (EEW) of 176 g mol^{-1}). Citric acid (CA), glutaric acid (GA), and pimelic acid (PA) were purchased from Sigma Aldrich. Glycerol triglycidyl ether (GTE) was kindly provided by Ipox Chemicals (Budapest, Hungary, EEW = 140-150 g mol^{-1}). All materials were used as received.

Thermoset preparation

Citric acid and either GA or PA diacid were mixed as solids in a glass flask before heating until a homogenous liquid was obtained, a slow heating rate was required to avoid bubble formation.* The acid mixture was then cooled to just above their crystallisation temperature (predetermined by DSC). Alongside ELO was preheated to the same temperature and then and mixed with a magnetic stirrer until the mixture appeared homogeneous, typically less than a minute. Preresin mixtures were poured into silicon moulds preheated to 100 °C on a hotplate and cured in a convection oven at 160 °C for 2 hours.

*Epoxy thermosets with no citric acid were synthesised by mixing ELO and diacid in a vial with a magnetic stirrer and heating until a homogeneous mixture was obtained, typically just below the diacid melting point.

Table S1. Overview of attempted thermoset formulations for glutaric acid (top) and pimelic acid (bottom). Successful compositions are highlighted in green. Red cells denote formulations where elevated viscosity and accelerated reaction kinetics hindered effective synthesis and moulding.

R_{tot} = 0.6		R_{tot} = 0.7		R_{tot} = 0.8	
R_{CA}	R_{GA}	R_{CA}	R_{GA}	R_{CA}	R_{GA}
0	0.6	0	0.7	0	0.8
0.1	0.5	0.1	0.6	0.1	0.7
0.2	0.4	0.2	0.5	0.2	0.6
0.3	0.3	0.3	0.4	0.3	0.5
0.4	0.2	0.35	0.35	0.4	0.4
0.45	0.15	0.4	0.3	0.5	0.3
0.5	0.1	0.5	0.2	0.55	0.25
0.6	0	0.55	0.15	0.6	0.2
		0.6	0.1	0.7	0.1
		0.7	0	0.8	0

R_{tot} = 0.6		R_{tot} = 0.7		R_{tot} = 0.8	
R_{CA}	R_{PA}	R_{CA}	R_{PA}	R_{CA}	R_{PA}
0	0.6	0	0.7	0	0.8
0.1	0.5	0.1	0.6	0.1	0.7
0.2	0.4	0.2	0.5	0.2	0.6
0.3	0.3	0.3	0.4	0.3	0.5
0.4	0.2	0.4	0.3	0.4	0.4
0.5	0.1	0.5	0.2	0.5	0.3
0.55	0.05	0.55	0.15	0.55	0.25
0.6	0	0.6	0.1	0.6	0.2
		0.6	0.1	0.7	0.1
		0.7	0	0.8	0

Differential scanning calorimetry (DSC)

Glass transition temperatures were conducted using a DSC25 DSC from TA Instruments (New Castle, DE, USA). Thermoset samples (2-10 mg) were placed in Tzero aluminium pans which were first cooled to -50°C and subsequently heated from -50°C to 275°C at 10°C min^{-1} under nitrogen gas. The glass transition temperature (T_g) was calculated as the slope change of the heat capacity plot.

Dynamic mechanical analysis (DMA)

Dynamic mechanical analysis was performed using a Mettler Toledo DMA1 instrument. Samples were run in a titanium compression clamp, on samples up to 10 mm in diameter. Measurements were conducted in single-frequency mode at 1 Hz. The temperature was ramped from –70 °C to 120 °C at a heating rate of 3 K min^{-1}. Storage modulus (E′), loss modulus (E″), and loss factor (tan δ) were recorded throughout the temperature sweep, and the maximum of E″ was taken as the Tg value.

Thermogravimetric analysis (TGA)

Thermal stability was determined using a TA Instruments TGA550 thermogravimeter. Samples (5 – 20 mg) were heated in platinum pans from 30°C to 600°C at 10°C min^{-1} under nitrogen gas at 10 mL min^{-1}. Weight loss was recorded as a function of temperature. Temperatures (°C) corresponding to 5% mass loss (T_{d5}) are reported.

Tensile strength

Tensile properties were measured using an Z50 Universal Testing Machine (AML Instruments, UK) equipped with a 1 kN load cell. Experiments were conducted according to ISO 527-1. Thermosets were cut out as Type 1BA, dog-bone samples, with a gauge length of 25 mm and a thickness of 2 mm. A minimum of 5 dog bones were tested *per* formulation, and the standard deviation and mean are reported. The samples were prestressed to 0.1 N and then loaded with a constant crosshead speed of 20 mm min^{-1}. We report tensile strength at break (σb, MPa), elongation at break (εb, %), Young's modulus (E, MPa) and Toughness (J/m^3).

Swell index and gel content

Samples (~100 - 200 mg) were carefully weighed into a vial and 30 mL of toluene was added. After sitting for 48 h at room temperature, the liquid phase was removed, and the mass of the swollen polymer was determined. The measurements for each type of material were done in triplicate, and the average is reported. Swell index (Q) was calculated from Eq. (4):

$$Q = (ms - mi) / mi \quad (4)$$

where ms and mi are the swollen and initial masses of the sample, respectively.

Gel content (GC, mass %) was determined by taking the insoluble fraction and drying under vacuum (50 mbar) at 50°C for 24 h. The measurements for each type of material were done in triplicate, and the average is reported. GC was calculated as follows from Eq. (5):

$$GC = mf/mi \times 100 \quad (5)$$

where mi and mf are the initial mass and mass after drying, respectively.

Degradation in alkaline solution

For evaluating the thermosets' chemical degradation, discs of epoxy thermoset 20 mm in diameter and 0.5 mm in thickness were immersed in an alkaline medium (1M NaOH) at room temperature and/or 60°C. The samples were then removed from the media at different time intervals, superficially dried with tissue paper, weighed, and then returned into the container. This procedure was repeated until sample dissolution or for seven days. Finally, the weight variation was plotted over time. All determinations were per formed in duplicate.

DSC thermograms

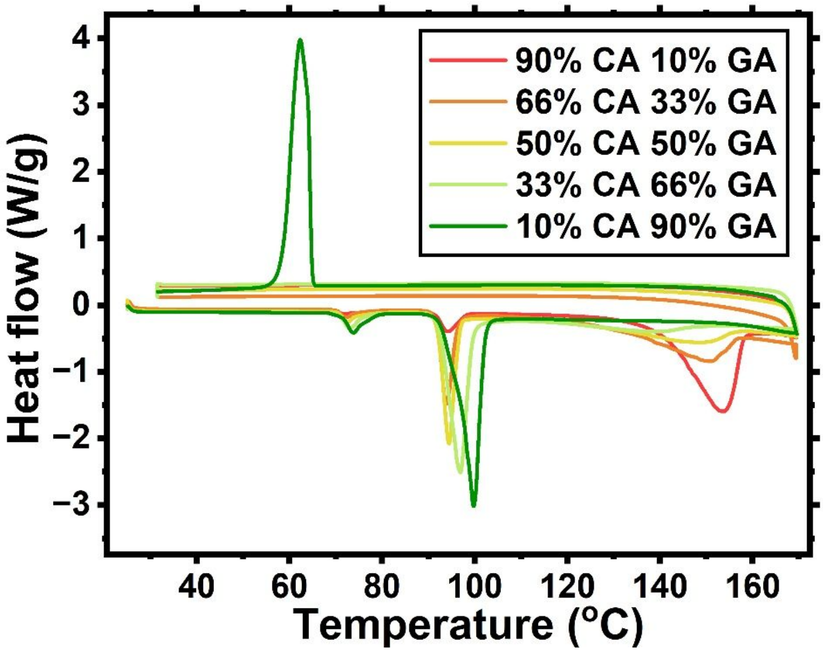

Figure S1: DSC thermograms of citric acid/glutaric acid mixtures showing no discernible crystallization peaks.

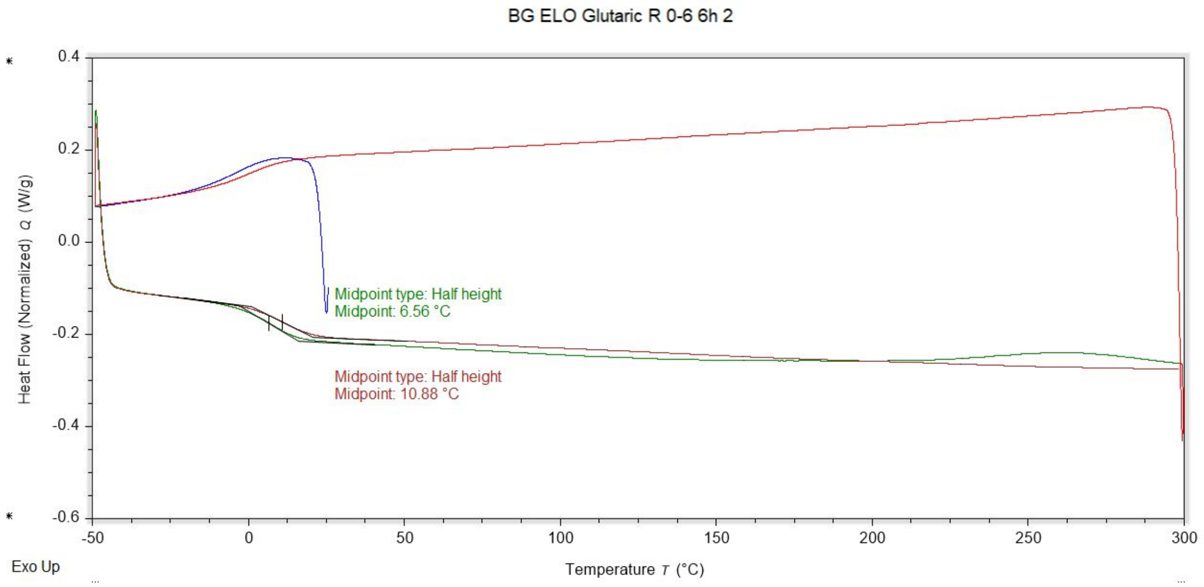

Figure S2: The DSC thermogram from the epoxy thermoset synthesized from GA/CA/ELO with R_{CA} = 0, R_{GA} = 0.6. First heating cycle in green, second in brown showing the increased T_g (6.56 to 10.88°C).

DMA plots

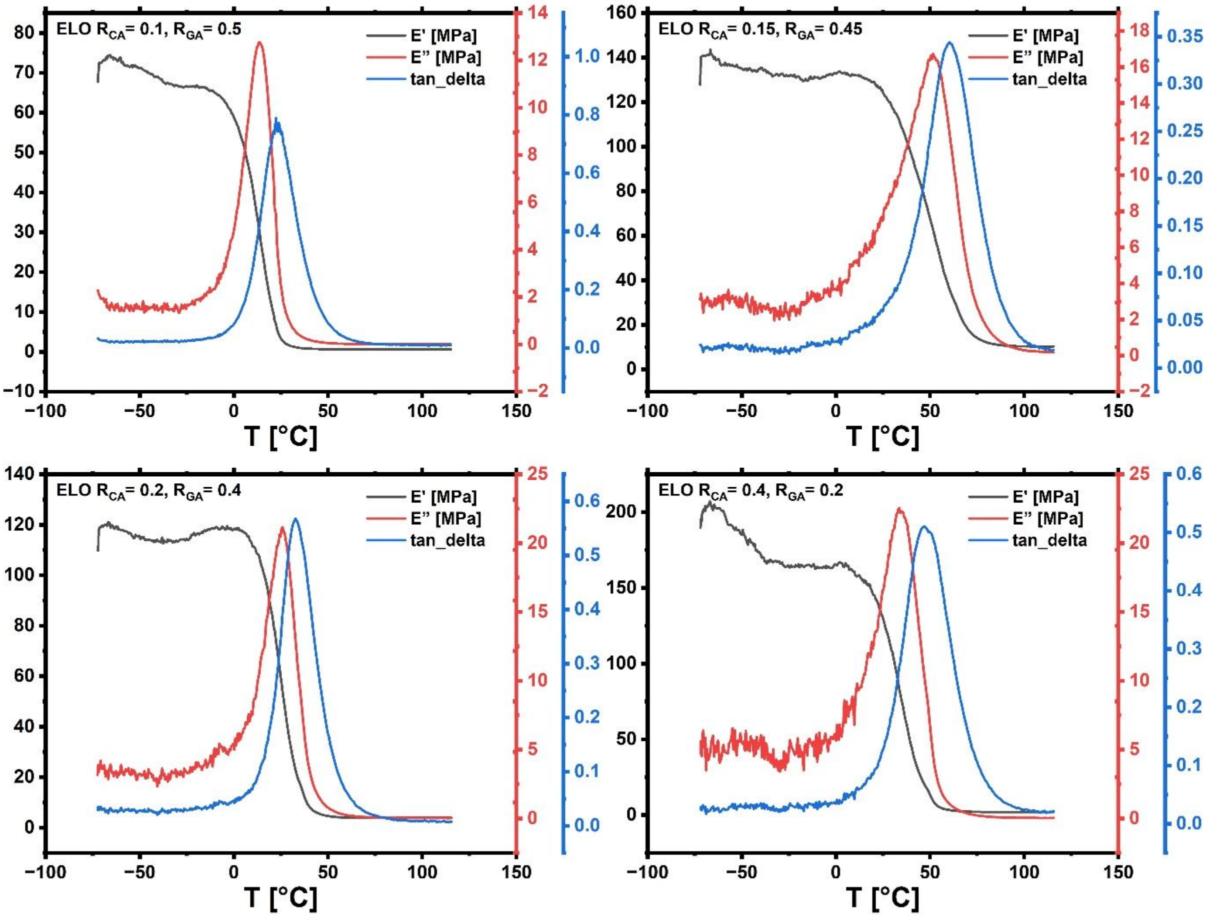

Figure S3: DMA plots for ELO thermosets with R_{tot} = 0.6 and R_{CA} values of 0.1 (top L), 0.15 (top R), 0.2 (bottom L) and 0.4 (bottom R).

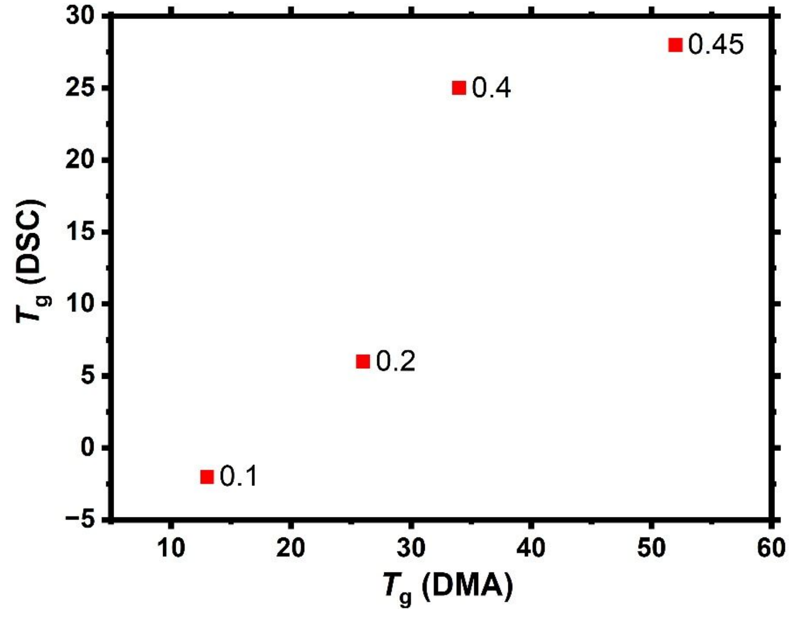

Figure S4: A plot comparing the T_g obtained from DMA and DSC with R_{tot} = 0.6 resins prepared with ELO, CA and GA. The R_{CA} value is depicted beside each data point.

TGA thermograms

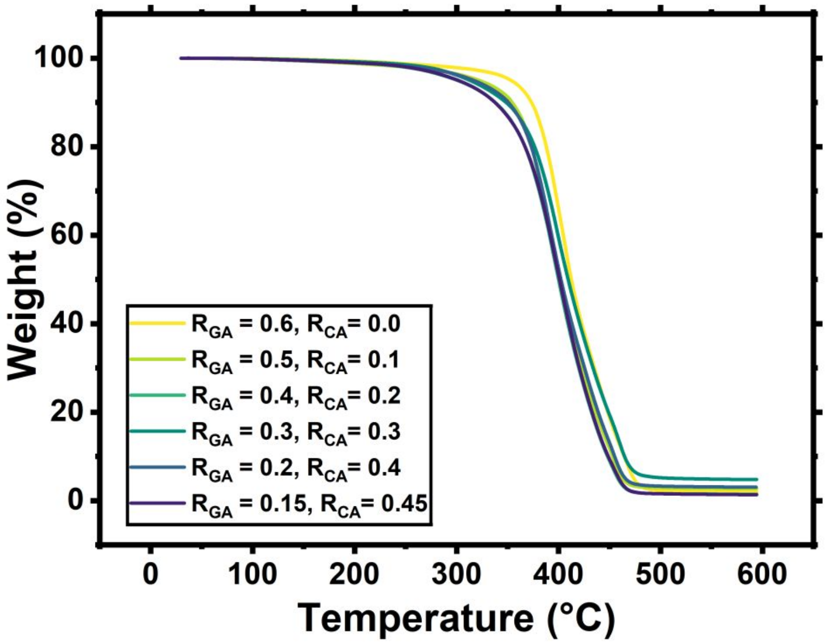

Figure S5. Thermal gravimetric analysis (TGA) thermograms for the CA/GA R_{tot} = 0.6 thermosets.

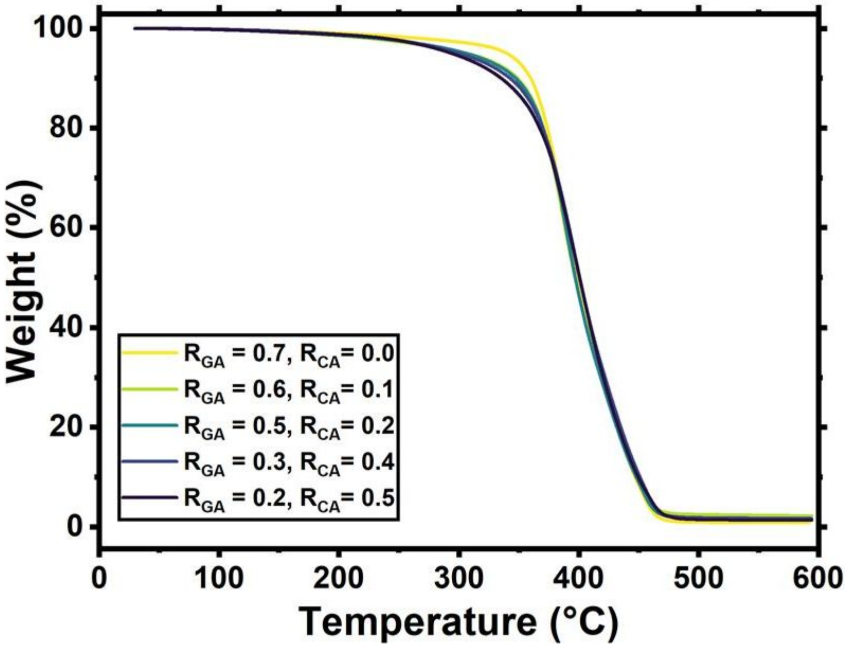

Figure S6. TGA thermograms for CA/GA R_{tot} = 0.7 thermosets.

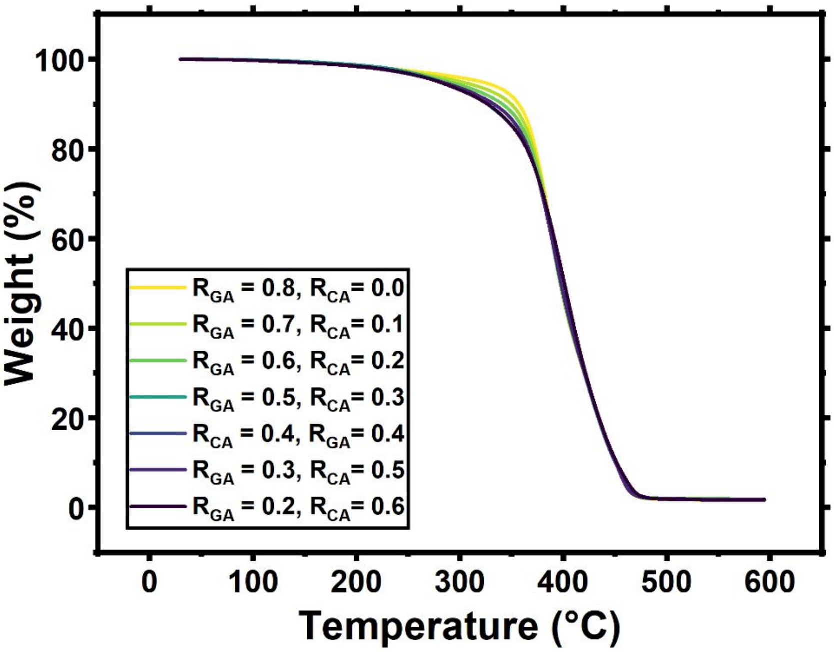

Figure S7. TGA thermograms for CA/GA R_{tot} = 0.8 thermosets.

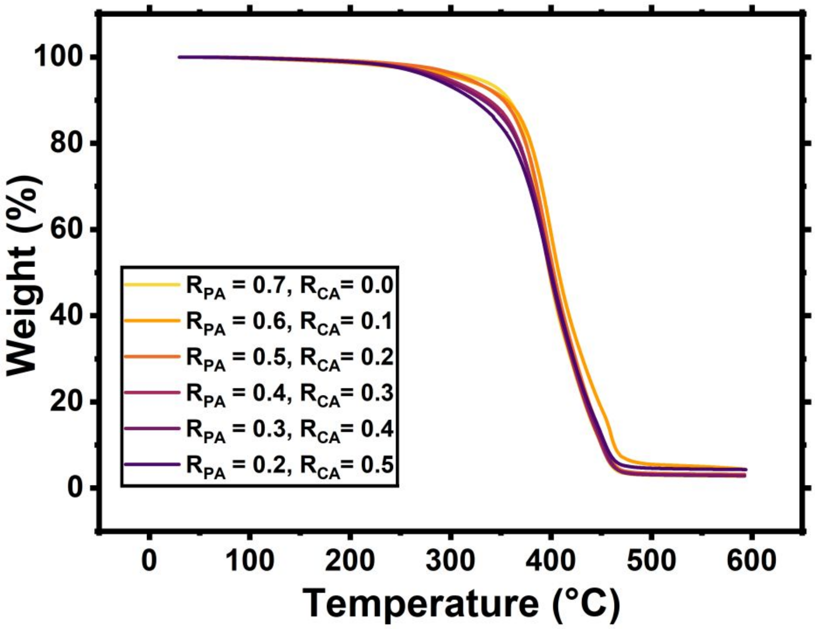

Figure S8. TGA thermograms for CA/PA R_{tot} = 0.6 thermosets.

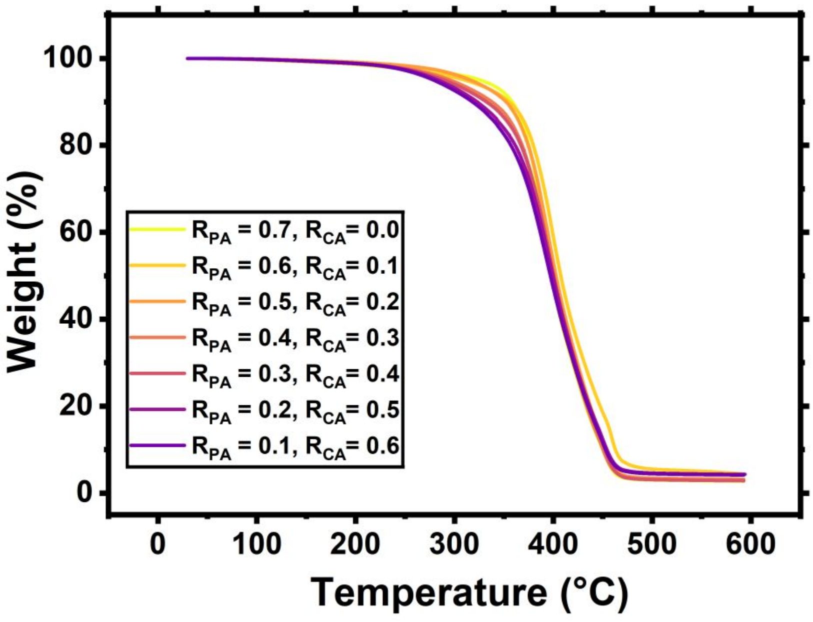

Figure S9. TGA thermograms for CA/PA $R_{tot} = 0.7$ thermosets.

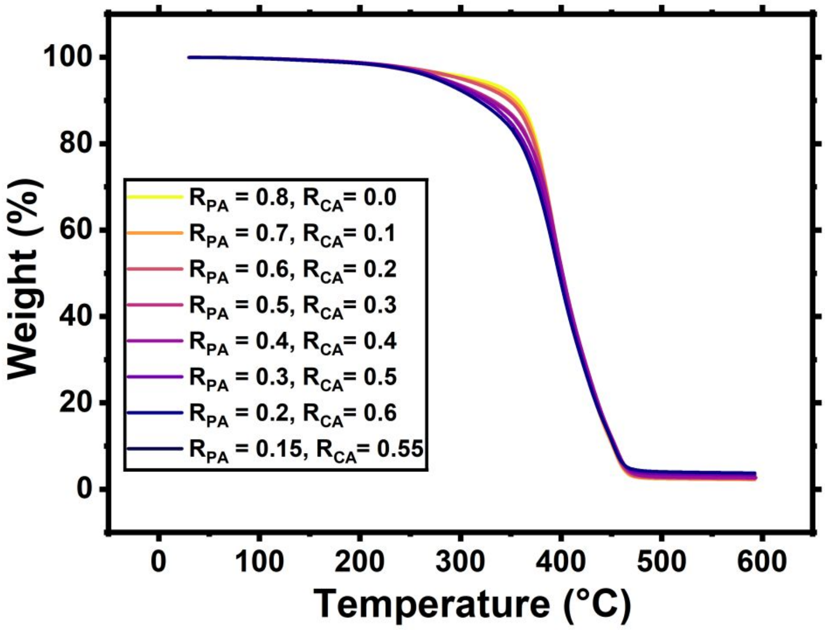

Figure S10. TGA thermograms for CA/PA $R_{tot} = 0.8$ thermosets.

Gel content of epoxy thermosets

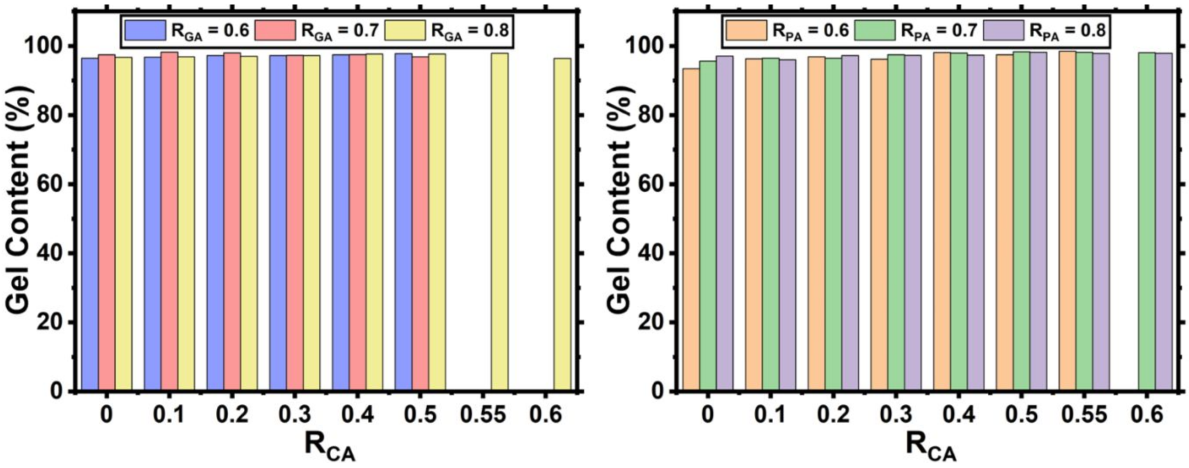

Figure S11: Gel content (%, in toluene) of the epoxy thermosets prepared from either glutaric acid (L) or pimelic acid (R) and citric acid at different R_{tot} values.

Degradation profile of CA/GA thermosets at room temperature

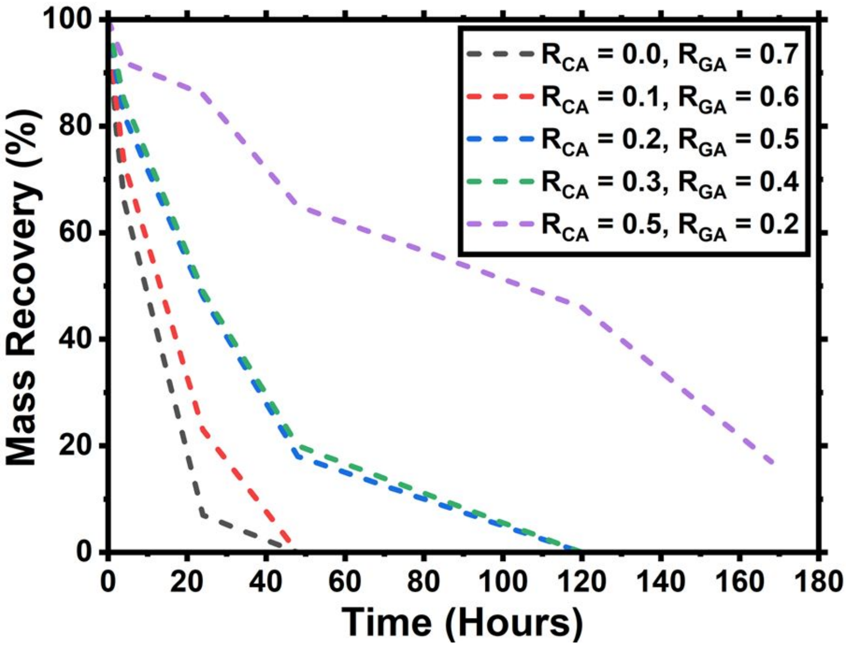

Figure S12: Degradation profile for CA/GA/ELO resins under alkaline conditions at room temperature over 168 hours.
